# Identification of specific protein amino acid substitutions of extended-spectrum β-lactamase (ESBL)-producing *Escherichia coli* ST131: a proteomics approach using mass spectrometry

**DOI:** 10.1038/s41598-019-45051-z

**Published:** 2019-06-12

**Authors:** Akihiro Nakamura, Masaru Komatsu, Yuki Ohno, Nobuyoshi Noguchi, Akira Kondo, Naoya Hatano

**Affiliations:** 1 Department of Clinical Laboratory Science, Faculty of Health Care, Tenri Health Care University, Tenri, Japan; 2 Department of Clinical Bacteriology, Clinical Laboratory Medicine, Tenri Hospital, Tenri, Japan; 3 The Integrated Center for Mass Spectrometry, Kobe University Graduate School of Medicine, Kobe, Japan

**Keywords:** Molecular medicine, Bacterial infection

## Abstract

The global pandemic of ESBL-producing *Escherichia coli* is associated with sequence type 131 (ST131). However, mechanisms of ST131 spread remain unclear. This study searched for proteins with amino acid substitutions specific for ST131 and used proteomics analysis to clarify ST131 characteristics. Five proteins had ST131-specific amino acid substitutions: uncharacterized protein YahO with E34A (*m/z* 7655); UPF0337 protein YjbJ with V59D, D60S and T63K (*m/z* 8351); uncharacterized protein YnfD with S106T (*m/z* 8448); and acid stress chaperone HdeA with Q92K and N94S (*m/z* 9714). Soluble cytochrome b562 (*m/z* 11783) showed seven amino acid substitutions, and the sequence differed between clade C of the pandemic clade and non-C. *In silico* analysis showed YahO protein-protein interaction with YjbJ, possibly related to biofilm formation. Although the function of soluble cytochrome b562 is electron transport of unknown function, its involvement in biofilm formation was predicted. HdeA was a gastric acid resistance-related protein. The function of YnfD was completely unclear. In conclusion, ST131-specific protein amino acid substitutions consisted mainly of a gastric acid resistance protein and proteins of unknown function (possibly involved in biofilm formation), which might be mechanisms for long-term colonization in the human intestinal tract.

## Introduction

Extended-spectrum β-lactamase (ESBL) and carbapenemase-producing multidrug-resistant *Enterobacteriaceae* have spread worldwide, mainly from *Escherichia coli* during the late 2000s^1–4^. Especially, ESBL *E*. *coli* have spread not only in hospitals but also in communities^3^. One of the causes of this spread is the global pandemic of *E*. *coli* sequence type (ST) 131 of multidrug-resistant *E*. *coli*^5,6^. ST131 is responsible for millions of global antimicrobial-resistant infections annually, for which the mortality rate is estimated to continue to exceed the current highest rate of malignant tumour mortality by 2050, if drug-resistant bacteria continue to increase without additional measures being taken^7^. The World Health Organization warned especially of multidrug-resistant *Enterobacteriaceae* such as *E*. *coli* in 2014^8^. Therefore, new strategies to prevent its spread are required in the future. Infections caused by ST131 are considered to be more pathogenic and refractory than those caused by non-ST131 because ST131 carries more drug-resistance factors and urinary tract virulence factors than non-ST131^1^.

Recently, the *fimH*30 subclone and O25b serotype of ST131 were found to participate significantly in fluoroquinolone resistance and virulence factors^9–11^. Within the *fimH*30 subclone, the *fimH*30-Rx subset often carries *bla*_CTX-M-15_^11^. Therefore, these clones show a higher prevalence for resistance to cephalosporins^2,9^. Moreover, the current pandemic clones are strains classified into the C clade by whole genome analysis^12^. Particularly, clades C1 and C2 are prevalent worldwide. In addition, the C1-M27 subclade with *bla*_CTX-M-27_ became especially prominent after 2009^13^. In this way, the population structure of ST131 has been explored extensively by many researchers. However, the reasons for the success of ST131 as an antimicrobial-resistant pandemic clone have not been clarified. The purpose of this study was to search for the proteins with specific amino acid substitutions for these clones using MALDI-TOF MS and identify them to clarify the characteristics of ST131 and its mechanisms of spread.

## Results

The search for specific proteins and their amino acid substitutions of ST131 was performed by MALDI-TOF MS analysis. Table 1 shows the results of the statistical analysis of the peaks of ST131 and non-ST131 using the Wilcoxon rank sum test of ClinProTools software. Ten peaks of *m/z* 3236, 4176, 4857, 5381, 6827, 7655, 8351, 8448, 9710 and 11783 were detected as reproducible specific peaks of ST131. The peaks of *m/z* 4176, 4857, 8351 and 8448 were detected 3 out of 3 times, whereas those of *m/z* 3236, 5381, 6827, 7655, 9710 and 11783 were detected 2 out of 3 times. The peaks of *m/z* 3236, 4176, 4857, 7655, 8351, 9710 and 11783 were also detected as ST131-specific peaks in the relevant published references.

**Table 1:** Results of statistical analysis of the peaks of ST131 and non-ST131 using Wilcoxon rank sum test of ClinProTools.

Specific peaks (*m/z*)	1st			2nd			3rd			References for ST131 peaks^a^
	P value	Ave. ST131	Ave. non-ST131	P value	Ave. ST131	Ave. non-ST131	P value	Ave. ST131	Ave. non-ST131	
3236	ND	ND	ND	0.0000528	4.14	3.02	0.00023	3.78	2.52	14
4176	0.0000026	5.32	3.96	0.0000701	8.99	6.33	<0.000001	6.94	4.23	20
4857	0.0168	4.79	4.32	0.000205	16.36	9.57	0.00000129	12.4	5.13	16
5381	ND	ND	ND	0.0195	26.56	23.7	0.0032	30.21	26.47	ND
6827	0.0000467	1.02	0.76	0.00015	2.44	1.94	ND	ND	ND	ND
7655	<0.000001	1.73	1.08	0.000234	2.67	2.22	ND	ND	ND	20
8351	<0.000001	10.39	6.66	0.000178	6.72	4.5	<0.000001	4.82	2.9	16, 20
8448	<0.000001	2.94	2.2	0.000405	1.75	1.49	0.0000498	1.45	1.22	ND
9710	ND	ND	ND	0.00044	14.84	8.64	0.000498	9.88	4.06	14, 15, 16
11783	<0.000001	0.91	0.5	<0.000001	0.9	0.62	ND	ND	ND	14, 16

ND, not detected; Ave., peak area/intensity average.

^a^Relevant published references for ST131-specific peaks.

### Identification of proteins with specific peaks in ST131 by LC-MS/MS

Table 2 shows the ST131 proteins with specific peaks identified using LC-MS/MS and their characteristics based on the Uniprot database. Five of the 10 specific peaks of ST131 were identified in this study. The *m/z* 7655 peak was identified as uncharacterized protein YahO (Uniprot accession no. P75694) belonging to the Bhs/McbA family. The chain domain of the YahO protein was DUF1471, and its function is unknown. The *m/z* 8351 peak was identified as UPF0337 protein YjbJ (Uniprot accession no. P68206) belonging to the UPF0337 (CsbD) family. The domain of YjbJ protein was CsbD, and its function is unknown. The *m/z* 8448 peak was identified as uncharacterized protein YnfD (Uniprot accession no. P76172), and its family was unclear. The chain domain of YnfD protein was DUF1161, and its function is also unknown. The *m/z* 9710 peak was identified as acid stress chaperone HdeA (Uniprot accession no. P0AES9) belonging to the HdeA family. The domain was HdeA, and the function is stress response protein acid-resistance protein. The *m/z* 11783 peak was identified as soluble cytochrome b562 (Uniprot accession no. P0ABE7) belonging to the cytochrome b562 family. The mature chain domain was cytochrome_B562, and the function is electron-transport of unknown function. The *m/z* 3236 and 4857 peaks were multivalent ions of *m/z* 9710 of HdeA, and the *m/z* 4176 peak was a multivalent ion of *m/z* 8351 of YjbJ. The *m/z* 5381 and 6827 peaks were not identified in this study.

**Table 2:** ST131-specific proteins identified using LC-MS/MS and their characteristics based on the Uniprot database.

Specific peaks (*m/z*)	Protein name	Predicted protein main localization^a^	Coding gene^a^	Family and domains^a^	Function^a^
3236	Acid stress chaperone HdeA (trivalent ion of 9710 *m/z*)	—	—		—
4176	UPF0337 protein YjbJ (divalent ion of 8351 *m/z*)	—	—		—
4857	Acid stress chaperone HdeA (divalent ion of 9710 *m/z*)	—	—		—
5381	Not identified	s/o cytosol^b^	—		—
6827	Not identified	s/o cytosol^b^	—		—
7655	Uncharacterized protein YahO	Periplasm	*yahO*	BhsA/McbA family, DUF1471	Unknown
8351	UPF0337 protein YjbJ	Cytosol	*yjbJ*	UPF0337 (CsbD) family, CsbD	Unknown (predicted stress-induced protein)
8448	Uncharacterized protein YnfD	s/o periplasm^b^	*ynfD*	Unknown, DUF1161	Unknown
9710	Acid stress chaperone HdeA	Periplasm	*hdeA*	HdeA family, HdeA	Stress response protein acid-resistance protein
11783	Soluble cytochrome b562	Periplasm	*cybC*	Cytochrome b562 family, cytochrome_B562	Electron-transport protein of unknown function

^a^The proteins were investigated for protein name, localization, coding gene, family and domain, and currently known function using the Uniprot database (http://www.uniprot.org).

^b^Unknown due to protein unidentified or not registered in the Uniprot database. Estimated from the fraction from which the proteins were extracted in this study.

### Comparison of amino acid sequences of identified proteins between ST131 and non-ST131

**Figure 1:**
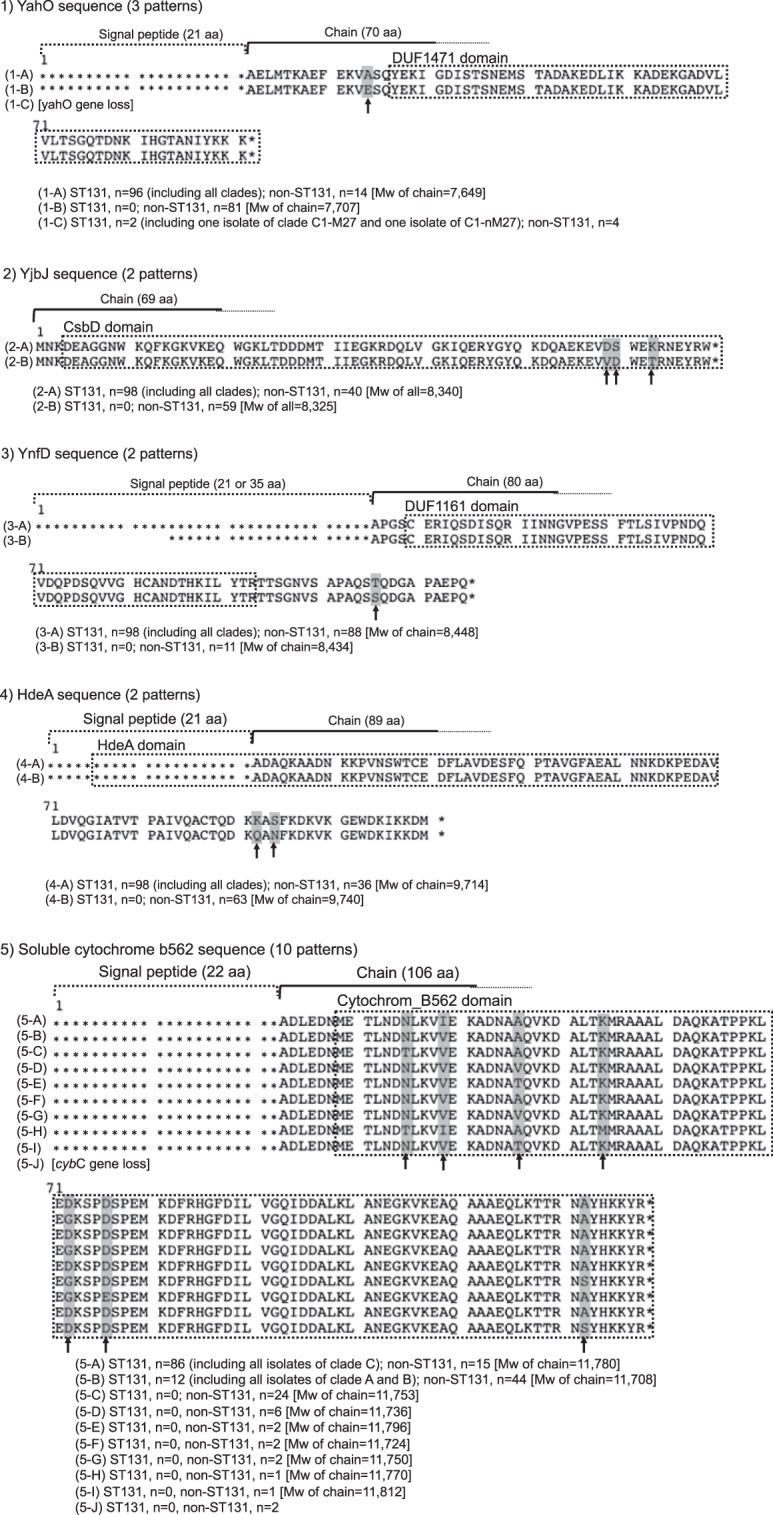
The amino acid sequence and its substitutions specific for ST131. The amino acid sequences of 1) uncharacterized protein YahO, 2) UPF0337 protein YjbJ, 3) uncharacterized protein YnfD, 4) acid stress chaperone HdeA and 5) soluble cytochrome b562 are shown.

Figure 1 shows the amino acid sequences and substitutions specific for ST131. The YahO-specific substitution of ST131 was E34A in the chain part outside the DUF1471 domain, and the rates were 98.0% (96/98) in ST131 including all clades and 14.1% (14/99) in non-ST131. The YjbJ-specific substitutions of ST131 were V59D, D60S and T63K in the chain part inside the CsbD domain, and the rates were 100% (98/98) in ST131 including all clades and 40.4% (40/99) in non-ST131. The YnfD-specific substitution of ST131 was S106T in the chain part outside the DUF1161 domain, and the rates were 100% (98/98) in ST131 including all clades and 88.9% (88/99) in non-ST131. The HdeA-specific substitutions of ST131 were Q92K and N94S in the chain part outside the HdeA domain, and the rates were 100% (98/98) in ST131 including all clades and 36.4% (36/99) in non-ST131. Moreover, we found seven amino acid substitutions specific for ST131 in the chain part inside the cytochrome_B562 domain of soluble cytochrome b562. In addition, the C1 and C2 subclades of the ST131 pandemic clade and the A and B clades of the ST131 non-pandemic clade differed in amino acid sequences of soluble cytochrome b562. The amino acid substitutions specific for the pandemic clade were T35N, V39I, VorT46A, M54K, G72D, E76D and S122A, which were possessed by all C clades. The amino acid substitutions most specific for ST131 were E34A of YahO followed by various substitutions of soluble cytochrome b562.

### *In silico* prediction of protein-protein interactions

**Figure 2:**
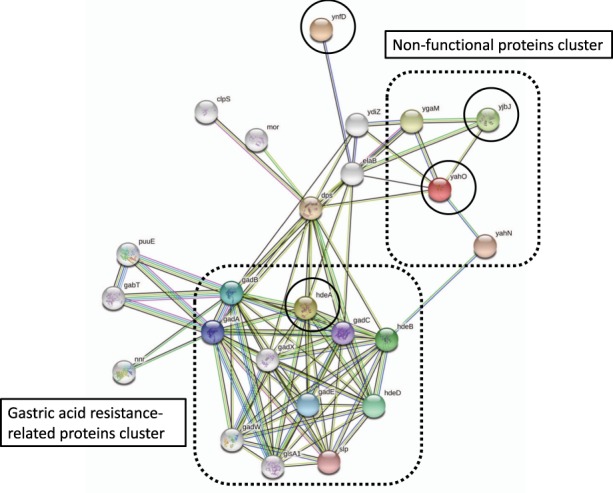
Protein-protein interaction of various proteins predicted using the STRING database (https://string-db.org). The organism database used *Escherichia coli* K12 MG1655. The STRING interaction map was generated using default settings (medium confidence of 0.400; criteria for linkages are neighbourhood, gene fusion, co-occurrence, co-expression, experiments, databases and text mining). The circles indicate the coding genes of proteins identified in this study.

**Figure 3:**
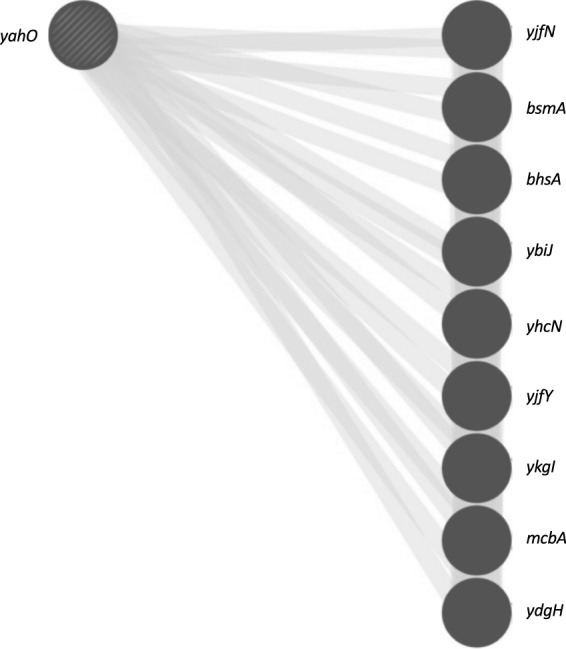
The results of the protein search with a domain structure common to YahO DUF1471 using GeneMANIA (https://genemania.org). The *bsmA* and *bhsA* are the coding genes of biofilm related proteins, and *mcbA* is the coding gene of colonic acid mucoidy stimulation protein. The *yjfN*, *ybiJ*, *yhcN*, *yjfY*, *ykgI* and *ydgH* are the coding genes of hypothetical proteins.

**Figure 4:**
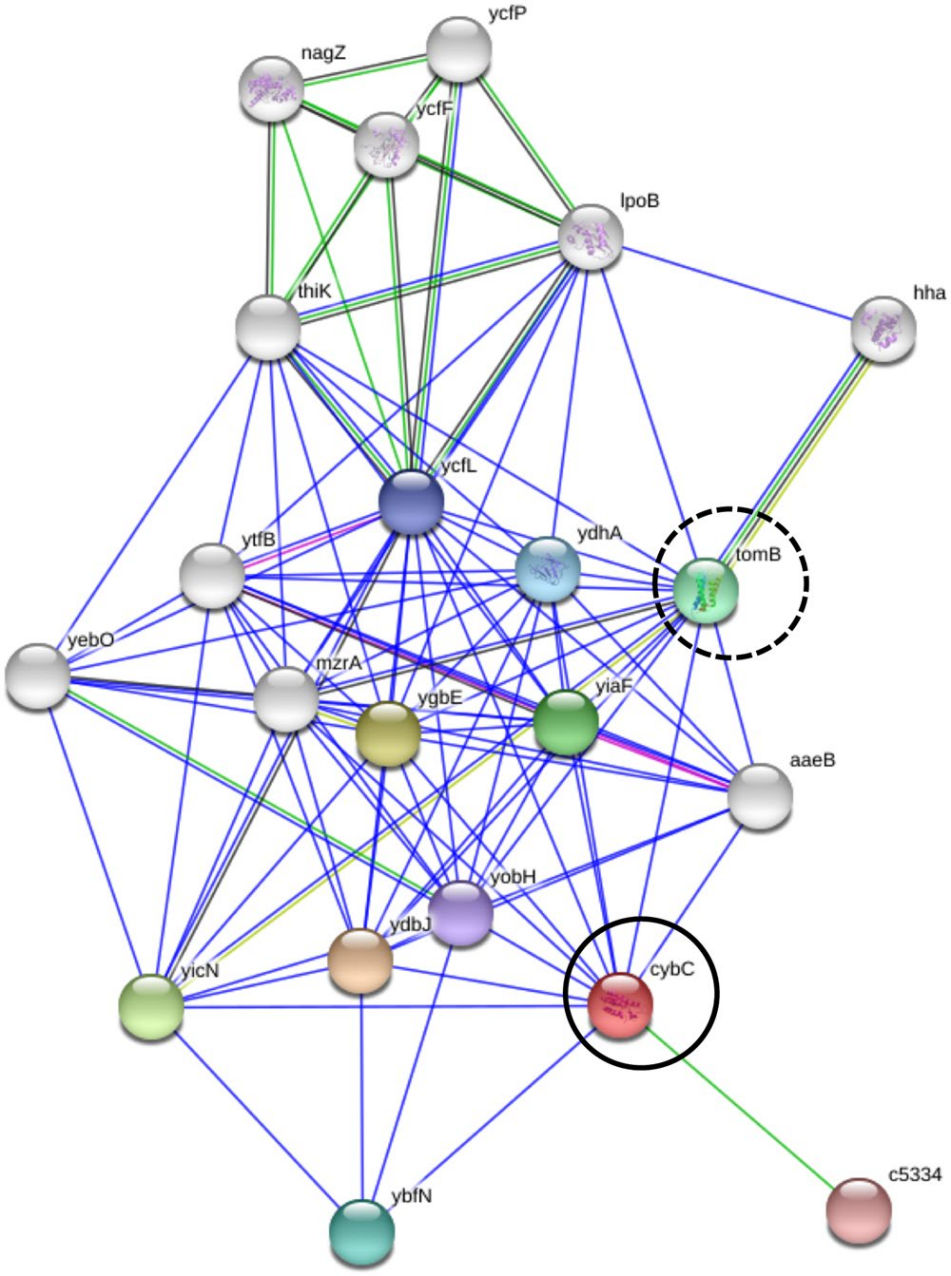
Protein-protein interaction of *cybC* coding soluble cytochrome b562 predicted using the STRING database (https://string-db.org). The organism database used *Escherichia coli* CFT073 from a urinary tract infection. The STRING interaction map was generated using default settings (medium confidence of 0.400; criteria for linkages are neighbourhood, gene fusion, co-occurrence, co-expression, experiments, databases and text mining). The circle indicates *cybC*, and the dashed-line circle indicates *tomB* showing interaction with *cybC*. The *tomB* is the coding gene of a biofilm formation regulator.

Figure 2 shows the identified proteins of ST131 predicted to have protein-protein interaction by the STRING database (organism database: *Escherichia coli* K12 MG1655). The *hdeA* was included in the cluster mainly composed of gastric acid resistance-related proteins such as *hdeB*, *gadA*, *gadB* and *gadC*. Besides, it was predicted that *yahO* and *yjbJ* might indicate protein-protein interaction, and their function was unknown, including that of its neighbouring proteins. The *ynfD* was a node isolated from those clusters. The *cybC* was not detected in this database. Figure 3 shows the results of a protein search with the domain structure common to YahO DUF1471 using GeneMANIA. The *bsmA* and *bhsA* have the DUF1471 domain as does YahO, and their function was related to biofilm formation. Furthermore, *mcbA* was colonic acid mucoidy stimulation protein, which was also a protein related to biofilm formation. Proteins common to the YjbJ domain were not searched. Figure 4 shows the predicted protein-protein interaction of *cybC* by the STRING database using *E*. *coli* CFT073 from a urinary tract infection. The *cybC* had an interaction with *tomB*, which is a biofilm formation regulator.

## Discussion

The purpose of this study was to search for and identify the proteins with amino acid substitutions specific for ST131 by proteomics analysis and to clarify the characteristics of ST131 to identify clear mechanisms for its spread. In this study, we found several proteins with the amino acid substitutions specific for ST131 and predicted their protein-protein interactions by bioinformatics using various web-based databases.

MALDI-TOF MS typing of ST131 has been explored extensively by many researchers. In a study using 149 ESBL-producing *E*. *coli* collected from seven acute-care hospitals in the Kyoto and Shiga regions of Japan, the most specific peak of ST131 was *m/z* 9720, with a sensitivity of 97.0% and a specificity of 91.5%^14^. Also, in a study using 109 ESBL-producing *E*. *coli* collected from clinical settings and the environment in France, the peak was identified as *m/z* 9713^15^. Moreover, in a study using 73 *E*. *coli* collected from different settings, geographic origins and isolation dates, several peaks of ST131 similar to those in the present study including *m/z* 9713 were detected^16^. However, no protein identification was performed in these previous studies. We also detected *m/z* 9710, which was similar to the peaks detected in these reports, and revealed it to be gastric acid resistance-related protein HdeA and found an amino acid substitution specific for ST131. HdeA localizes in the bacterial periplasmic space and exhibits acid resistance by expressing chaperone-like activity that suppresses denaturation of proteins under acidic conditions. Although it is unclear how this amino acid substitution affects the strains, ST131 might have high resistance to gastric acid that allows it to reach the human intestinal tract.

In this study, we found YahO protein showing a *m/z* 7650 peak that is more specific than HdeA. We already found in our earlier study that YahO is a protein specific for ST131^17^. We predicted its function using the STRING and GeneMANIA databases, which predicted YahO to have protein-protein interaction with YjbJ, another protein we found, suggesting that they might be related to biofilm formation. Kudinha *et al*.^18^ and Clermont *et al*.^19^ both found that the prevalence of strains producing biofilms was greater among ST131 than among non-ST131 clinical strains. Besides, Eletsky *et al*.^20^ researched the function of the DUF1471 domains of *Salmonella* protein YahO, and they concluded that YahO is closely related to YcfR/BhsA, YbiM/McbA, YjfO/BsmA or YcfR, which are associated with a stress response and biofilm formation. This was also predicted in our study. In the future, it will be necessary to elucidate how this specific amino acid substitution of YahO and YjbJ affects biofilm formation. In addition, in the *cybC* sequence of soluble cytochrome b562, we found a specific sequence in the pandemic clade C. Moreover, *cybC* had an interaction with *tomB*, which is a biofilm formation regulator. These proteins possibly involved in biofilm formation may be related to the affinity of ST131 for humans.

The characteristics of ST131 clarified in this study might allow a hypothesis of the mechanism of colonization in the human intestinal tract. We hypothesized that ST131 does not die when passing through the stomach because of its resistance to gastric acid, and when it reaches the intestinal tract, it survives for a long time by forming a biofilm in the intestinal tract. As this is only a hypothesis, it will be necessary to elucidate the influence of these amino acid substitutions in the future.

This study has several limitations. First, the ST131 and non-ST131 strains used in this study were collected only from various regions in Japan and not sites worldwide. However, the strains were collected from all over Japan and were not localized to specific regions. Because the strain characteristics, such as *fimH*30 typing and the MALDI data, are consistent with those of other previous studies, we believe the findings are reliable. Second, as stated previously, although we clarified the difference in amino acid substitutions in various proteins between ST131 and non-ST131, the influence of these substitutions remains unclear. In the future, we will perform function analysis, such as pull-down and genome editing assays, of these proteins. Finally, although YnfD was identified as a protein with an amino acid substitution specific for ST131, its function could not be deduced at all.

In conclusion, we found several proteins with amino acid substitutions specific for ST131. They consisted mainly of gastric acid resistance proteins and proteins with unknown function (but which were estimated to be biofilm formation proteins). These proteins might be associated with the mechanism of long-term colonization of ST131 in the human intestinal tract. In the future, by clarifying the functions of these proteins and their substitutions, the mechanism behind the worldwide pandemic of the ST131 might be further clarified.

## Materials and Methods

### Bacterial isolates

To search for proteins with specific amino acid substitutions for ST131, 197 ESBL-producing *E*. *coli* (97 strains of ST131 and 100 strains of non-ST131) isolated from clinical specimens collected at 24 clinical facilities in Japan between 2011 to 2013 were used. The 24 clinical facilities comprised 23 acute-care hospitals and 1 commercial laboratory located throughout Japan: 18 facilities from Western Japan and 6 facilities from Eastern Japan. These samples, which were derived from our previous study^17^ (n = 74) and the present study (n = 123), were kindly provided by the Association of Japan Community Healthcare Organization (JCHO) hospitals and the Study of Bacterial Resistance in the Kinki Region of Japan (SBRK) and were randomly extracted (Table 3).

**Table 3:** Characteristics of the O25b, *fimH*30 and ESBL genotypes in ESBL-producing *E. coli* used in this study

Sequence type	ST131 clade	ST131 subclade	O serotypes and *fimH* types	ESBL gene
ST131 (97)^a^	C (85)	C2 (34)	O25b, *fimH*30Rx (34)	CTX-M-1 group [M-15] (33), M-1 [M-15] + M-9 group (1)
		C1-M27 (33)	O25b, *fimH*30 non-Rx (30) O25b and O16 negative, *fimH*30 non-Rx (3)	CTX-M-9 group (29), M-1 group [M-15] (1) CTX-M-9 group (3)
		C1-nM27 (18)	O25b, *fimH*30 non-Rx (16) O25b and O16 negative, *fimH*30 non-Rx (2)	CTX-M-9 group (10), M-2 group (5), M-1 group [M-15] (1) CTX-M-9 group (2)
	A (7)	—	O16, *fimH*30 negative (5) O25b, *fimH*30 negative (1) O25b and O16 negative, *fimH*30 negative (1)	CTX-M-9 group (4), M-1 group [non-M-15] (1) CTX-M-9 group (1) CTX-M-9 group (1)
	B (5)	—	O25b, *fimH*30 negative (5)	CTX-M-2 group (2), M-1 group [non-M-15] (2), SHV-12 (1)
Non-ST131 (100)	—	—	—	CTX-M-9 group (45), M-1 group (39), M-2 group (11), SHV-12 (5), SHV-2 (1)

^a^Numbers in parentheses indicate the number of samples.

These strains were characterized by ST131 typing, ST131 clade typing, O25b serotyping, *fimH*30 subclonal typing and β-lactamase gene typing. Bacterial DNA was purified using a QIAmp DNA Mini Kit (QIAGEN, Hilden, Germany). In ST131 typing, ST131 was defined based on PCR detection of ST131-specific SNPs in the *mdh* and *gyrB* alleles^21^. In addition, the ST131 clade and subclade were defined based on multiplex PCR using seven specific SNP primers^22^. In O25b serotyping and *fimH*30 subclone typing, specific PCR detection based on each specific primer was performed^9,23^. In addition, *fimH30*-Rx was defined based on PCR detection of *ybbW* SNP typing^24^. In β-lactamase gene typing, strains were analysed to determine the presence of ESBL encoded by *bla*_SHV_, *bla*_TEM_, *bla*_CTX-M-1_-like, *bla*_CTX-M-2_-like, *bla*_CTX-M-8_-like and *bla*_CTX-M-9_-like^25,26^. In addition, PCR direct sequencing analysis was performed on *bla*_SHV_- and *bla*_TEM_-positive strains^27^.

### MALDI-TOF MS data analysis

The method of MALDI-TOF MS analysis followed that of our previous study^17^. The strains were cultured at 37 °C for 16-24 h using 5% sheep blood agar. We performed ethanol-formic acid protein extraction from grown colonies for preparation of the MALDI-TOF MS analysis and used Bruker Bacterial Test Standard (Bruker Daltonik, Bremen, Germany) for calibration. MALDI-TOF MS analysis was performed using MALDI Biotyper (Bruker Daltonik). Spectra obtained by MALDI-TOF MS analysis were used for comparison of the spectrum of the ST131 with that of the non-ST131 using ClinProTools v2.2 (Bruker Daltonik), and peaks specific to ST131 were searched. We analysed spectra with Peak Statistic Calculation, which includes the Wilcoxon rank sum test. We analysed each isolate three times, and the peaks that were observed at least two of the three times in Peak Statistic Calculation were extracted as reproducible specific peaks of ST131. For the specific peaks judged to be significant by ClinProTools, peaks within the range of ±400 ppm were confirmed by FlexAnalysis v3.4 software (Bruker Daltonik) for each strain. The settings used for MALDI-TOF MS analysis were linear positive mode, 20-Hz laser frequency, 20-kV acceleration voltage, 18.5-kV IS2 voltage, 250-ns extraction delay, and 2000–20,000 *m/z* range.

### Proteomic analysis

The identification of the proteins with mass peaks specific to ST131 followed that of our previous study^17^. The strains with these specific peaks were incubated using 10 L LB broth, and then all pellets were collected after centrifugal separation. Next, to collect periplasmic proteins, the pellets were suspended with 30 mM Tris-HCl pH 8.0 with 20% sucrose on ice and incubated for 10 min at room temperature with slow mixing. Then, after centrifugal separation, the pellets underwent osmotic shock for 10 min using 5 mM MgSO_4_ solution on ice. After centrifugal separation, the supernatant was used as the periplasmic proteins fraction after being dialyzed against 20 mM Tris-HCl pH 8.0. The pellets were suspended with 20 mM Tris-HCl pH 8.0, and soluble cytoplasmic proteins were collected after sonication of the pellets for 15 min. After centrifugal separation, the supernatant was used as the soluble cytoplasmic proteins fraction after being dialyzed against 20 mM Tris-HCl pH 8.0. Then, each of the protein fractions was purified by ion-exchange chromatography using Macro-Prep diethylaminoethyl support (Bio-Rad Laboratories, Inc., Hercules, CA) and reversed-phase HPLC using TSKgel ODS-100V (Tosoh Corporation, Tokyo, Japan). Finally, the gel obtained by tricine–SDS-PAGE with silver staining was analysed by LC-MS/MS using a High-Performance Liquid Chromatograph Ion-trap Time-of-Flight mass spectrometer (Shimazu Corporation, Kyoto, Japan) after in-gel digestion with trypsin of the target protein band was conducted. Trypsin digestion was performed using 10 µg/mL of sequencing grade modified trypsin (Promega KK, Tokyo, Japan), and the trypsin solution was incubated at 37 °C for 15 h. Spectra obtained from bottom-up proteomics were analysed using Mascot MS/MS Ion Search (Matrix Science K.K., Tokyo, Japan) to search and assign the obtained peptides to the SwissProt database. The identified proteins were investigated for protein name, localization, coding gene, family and domain, and currently known function using the Uniprot database (http://www.uniprot.org).

### Genomic analysis

From the amino acid sequencing identified by LC-MS/MS, the forward and reverse primers of each target protein were designed (Table S1), and comparison of amino acid sequences translated from nucleotide sequences between ST131 and non-ST131 was performed by PCR direct sequencing. Seaview software version 4 (http://pbil.univ-lyon1.fr/software/seaview) was used for sequence alignment and comparison. Also, the theoretical molecular weight calculated from the amino acid sequences was computed using the Compute pI/Mw tool (http://web.expasy.org/compute_pi/).

### *In silico* prediction of protein-protein interactions

The ST131 proteins predicted to have protein-protein interaction were identified by STRING database version 10.5 (https://string-db.org). Furthermore, the proteins whose functions were not predicted on STRING were predicted by investigating protein domain common proteins using GeneMANIA (https://genemania.org).

## Supplementary information

Supplementary Table

## References

[1] Nicolas-Chanoine MH, Bertrand X, Madec JY (2014). *Escherichia coli* ST131, an intriguing clonal group. Clin. Microbiol. Rev..

[2] Mathers AJ, Peirano G, Pitout JR (2015). The role of epidemic resistance plasmids and international high-risk clones in the spread of multidrug-resistant Enterobacteriaceae. Clin. Microbiol. Rev..

[3] Nakamura A (2016). Analysis of molecular epidemiologic characteristics of extended-spectrum β-lactamase (ESBL)-producing *Escherichia coli* colonizing feces in hospital patients and community dwellers in a Japanese city. J. Infect. Chemother.

[4] Ohno Y (2017). Molecular epidemiology of carbapenemase-producing Enterobacteriaceae in a primary care hospital in Japan, 2010-2013. J. Infect. Chemother.

[5] Rogers BA, Sidjabat HE, Paterson DJ (2011). *Escherichia coli* O25b-ST131: a pandemic, multiresistant, community-associated strain. J. Antimicrob. Chemother..

[6] Matsumura Y (2012). Emergence and spread of B2-ST131-O25b, B2-ST131-O16 and D-ST405 clonal groups among extended-spectrum-β-lactamase-producing *Escherichia coli* in Japan. J. Antimicrob. Chemother..

[7] O’Neill J. The review on antimicrobial resistance: tackling drug-resistant infections globally: final report and recommendations. HM Government and Welcome Trust, UK (2016).

[8] World Health Organization. Antimicrobial resistance: global report on surveillance (2014).

[9] Colpan A (2013). *Escherichia coli* sequence type 131 (ST131) subclone H30 as an emergent multidrug-resistant pathogen among US veterans. Clin. Infect. Dis.

[10] Johnson, J. R. *et al*. Abrupt emergence of a single dominant multidrug-resistant strain of *Escherichia coli*. *J*. *Infect*. *Dis*. **207**, 919–928 (2013). 10.1093/infdis/jis933 PMC3571447 23288927

[11] Price LB (2013). The epidemic of extended-spectrum-β-lactamase-producing *Escherichia coli* ST131 is driven by a single highly pathogenic subclone, H30-Rx. mBio..

[12] Petty NK (2014). Global dissemination of a multidrug resistant *Escherichia coli* clone. Proc. Natl. Acad. Sci. USA..

[13] Matsumura Y (2016). Global *Escherichia coli* sequence type 131 clade with bla_CTX-M-27_ gene. Emerg. Infect. Dis..

[14] Matsumura Y (2014). Detection of extended-spectrum-β-lactamase-producing *Escherichia coli* ST131 and ST405 clonal groups by matrix-assisted laser desorption ionization-time of flight mass spectrometry. J. Clin. Microbiol..

[15] Lafolie J, Sauget M, Cabrolier N, Hocquet D, Bertrand X (2015). Detection of *Escherichia coli* sequence type 131 by matrix-assisted laser desorption ionization time-of-flight mass spectrometry: implications for infection control policies?. J. Hosp. Infect..

[16] Novais Â (2014). MALDI-TOF mass spectrometry as a tool for the discrimination of high-risk *Escherichia coli* clones from phylogenetic groups B2 (ST131) and D (ST69, ST405, ST393). Eur. J. Clin. Microbiol. Infect. Dis..

[17] Nakamura A (2015). Rapid detection of B2-ST131 clonal group of extended-spectrum β-lactamase-producing *Escherichia coli* by matrix-assisted laser desorption ionization-time-of-flight mass spectrometry: discovery of a peculiar amino acid substitution in B2-ST131 clonal group. Diagn. Microbiol. Infect. Dis..

[18] Kudinha T (2013). *Escherichia coli* sequence type 131 as a prominent cause of antibiotic resistance among urinary *Escherichia coli* isolates from reproductive-age women. J. Clin. Microbiol..

[19] Clermont O (2008). The CTX-M-15-producing *Escherichia coli* diffusing clone belongs to a highly virulent B2 phylogenetic subgroup. J. Antimicrob. Chemother.

[20] Eletsky A (2014). Structural and functional characterization of DUF1471 domains of *Salmonella* proteins SrfN, YdgH/SssB, and YahO. PLoS. One..

[21] Johnson JR (2009). Epidemic clonal groups of *Escherichia coli* as a cause of antimicrobial-resistant urinary tract infections in Canada, 2002 to 2004. Antimicrob. Agents. Chemother..

[22] Matsumura Y, *et al*. Rapid identification of different *Escherichia coli* sequence type 131 clades. *Antimicrob*. *Agents*. *Chemother*. **61**, pii: e00179–17 (2017). 10.1128/AAC.00179-17 PMC5527616 28584160

[23] Clermont O (2008). The CTX-M-15-producing *Escherichia coli* diffusing clone belongs to a highly virulent B2 phylogenetic subgroup. J. Antimicrob. Chemother.

[24] Banerjee Ritu, Robicsek Ari, Kuskowski Michael A., Porter Stephen, Johnston Brian D., Sokurenko Evgeni, Tchesnokova Veronika, Price Lance B., Johnson James R. (2013). Molecular Epidemiology of Escherichia coli Sequence Type 131 and Its H30 and H30-Rx Subclones among Extended-Spectrum-β-Lactamase-Positive and -Negative E. coli Clinical Isolates from the Chicago Region, 2007 to 2010. Antimicrobial Agents and Chemotherapy.

[25] Yagi T (1997). Nosocomial spread of cephem-resistant *Escherichia coli* strains carrying multiple Toho-1-like β-lactamases genes. Antimicrob. Agents. Chemother..

[26] Sabaté M (2000). Cloning and sequence of the gene encoding a novel cefotaxime-hydrolyzing beta- lactamase (CTX-M-9) from *Escherichia coli* in Spain. Antimicrob. Agents. Chemother..

[27] Jeong SH (2005). Dissemination of transferable CTX-M-type extended-spectrum beta-lactamase-producing *Escherichia coli* in Korea. J. Appl. Microbiol..

