# Supplementary material for: Identification of specific protein amino acid substitutions of extended-spectrum β-lactamase (ESBL)-producing Escherichia coli ST131: a proteomics approach using mass spectrometry
Source: Sci Rep. 2019 Jun 12;9:8555. doi: 10.1038/s41598-019-45051-z (PMC6561927; doi:10.1038/s41598-019-45051-z)
Supplement: Supplementary file 1 — Supplementary Table [file 41598_2019_45051_MOESM1_ESM.docx]

**Supplementary Information for**

**Identification of specific protein amino acid substitutions of extended-spectrum β-lactamase (ESBL)-producing *Escherichia coli* ST131: a proteomics approach using mass spectrometry**

Akihiro Nakamura^1*^, Masaru Komatsu^1^, Yuki Ohno^2^, Nobuyoshi Noguchi^1,2^, Akira Kondo^1^, Naoya Hatano^3^

^1^Department of Clinical Laboratory Science, Faculty of Health Care, Tenri Health Care University, Tenri, Japan

^2^Department of Clinical Bacteriology, Clinical Laboratory Medicine, Tenri Hospital, Tenri, Japan

^3^The Integrated Center for Mass Spectrometry, Kobe University Graduate School of Medicine. Kobe, Japan

Corresponding author: Akihiro Nakamura

Department of Clinical Laboratory Science, Faculty of Health Care, Tenri Health Care University, Tenri, Japan, 80-1 Bessho-cho, Tenri, Nara 632-0018, Japan

Phone: +81-743-63-7811; Fax: +81-743-63-6211; E-mail: a.nakamura@tenriyorozu-u.ac.jp

**Supplementary Table**

**Table S1.** Primers used for detection of the amino acid substitution of each identified protein

| Identified protein name | Coding  gene name | Primer sequence (5'-3') | Product size (bp) |
| --- | --- | --- | --- |
| YahO | *yahO* | Forward: ATGAAAATAATCTCTAAAATGTTAG Reverse: TTACTTCTTCTTATAAATATTTGCCG | 273 |
| YjbJ | *yjbJ* | Forward: ATGAATAAAGATGAAGCCGG Reverse: TTACCAGCGATATTCATTGC | 207 |
| YnfD | *ynfD* | Forward: ATGATGAACTCTCAACCTG Reverse: TTACTGCGGTTCGGCAGGC | 303 or 345 |
| HdeA | *hdeA* | Forward: ATGAAAAAAGTATTAGGCG Reverse: TTACATATCTTTCTTAATTTTG | 330 |
| Soluble cytochrome b562 | *cybC* | Forward: ATGCGTAAAAGCCTGTAGC Reverse: TTAACGATACTTCTGGTGATAG | 340 |
